# The Burden of Severely Drug-Refractory Epilepsy: A Comparative Longitudinal Evaluation of Mortality, Morbidity, Resource Use, and Cost Using German Health Insurance Data

**DOI:** 10.3389/fneur.2017.00712

**Published:** 2017-12-22

**Authors:** Adam Strzelczyk, Claudia Griebel, Wolfram Lux, Felix Rosenow, Jens-Peter Reese

**Affiliations:** ^1^Epilepsy Center Frankfurt Rhine-Main, Department of Neurology, Goethe University, Frankfurt am Main, Germany; ^2^Epilepsy Center Hessen, Department of Neurology, Philipps-University Marburg, Marburg, Germany; ^3^LivaNova Deutschland GmbH, München, Germany; ^4^HGC GesundheitsConsult GmbH, Düsseldorf, Germany; ^5^Institute of Health Service Research and Clinical Epidemiology, Coordinating Center for Clinical Trials, Philipps-University Marburg, Marburg, Germany

**Keywords:** seizure, morbidity, epidemiology, population-based, secondary data analysis

## Abstract

**Purpose:**

To evaluate long-term outcome of three years and treatment patterns of patients suffering from severely drug-refractory epilepsy (SDRE).

**Methods:**

This analysis was population-based and retrospective, with data collected from four million individuals insured by statutory German health insurance. ICD-10 codes for epilepsy (G40*) and intake of anticonvulsants were used to identify prevalent cases, which were then compared with a matched cohort drawn from the population at large. Insurance data were available from 2008 to 2013. Any patient who had been prescribed with at least four different antiepileptic drugs (AEDs) in an 18-month period was defined as an SDRE case.

**Results:**

A total of 769 patients with SDRE were identified. Of these, 19% were children and adolescents; the overall mean age was 42.3 years, 45.4% were female and 54.6% male. An average of 2.7 AEDs per patient was prescribed during the first follow-up year. The AEDs most commonly prescribed were: levetiracetam (53.5%), lamotrigine (41.4%), valproate (41.3%), lacosamide (20.4%), and topiramate (17.8%). During 3-year follow-up, there was an annual rate of hospitalization in the range 42.7 to 55%, which was significantly higher than the 11.6–12.8% (*p* < 0.001) for the matched controls. Admissions to hospital because of epilepsy ranged between 1.7 and 1.9 per year, with an average duration for each epilepsy-caused hospitalization of 10–11.1 days. The number of comorbidities for SDRE patients was significantly increased compared with the matched controls: depression (28% against 10%), vascular disorders (22% against 5%), and injury rates were also higher (head 16% against 3%, trunk and limbs 16% against 8%). The 3-year mortality rate for SDRE patients was 14% against 2.1% in the matched cohort.

**Conclusion:**

SDRE patients are treated with AED polytherapy for all of the 3-year follow-up period. They are hospitalized more frequently than the general population and show increased morbidity levels and a sevenfold increase in mortality rate over 3 years. Further examination is required of ways in which new approaches to treatment could lead to better outcomes in severely affected patients.

## Introduction

Epilepsy, a chronic neurological disorder, is not only common, but also burdensome both for individuals and for society. First diagnosis triggers costs in regard to diagnostic procedures, inpatient admission, and patient’s loss of income (1, 2). Patients may experience social stigma, have restricted employment opportunities, and suffer impairment to the quality of life of both themselves and their caregivers. Subsequently, indirect and intangible costs result as early as the first seizure or the first diagnosis occurs (3–9).

For most patients, treatment with anticonvulsants will be necessary over a long period, and—despite optimal medical treatment—up to 30% of patients will incur refractory (10–13). Due to the high amount of total costs associated with a refractory course of disease, such drug-refractory epilepsy requires sound economic evaluation (14–19). In addition, uncontrolled seizures are often accompanied with an increased risk of psychiatric comorbidities, such as depression, and an increase in morbidity, such as falls and injuries as direct consequence of seizures, as well as an increased overall mortality from sudden unexpected death in epilepsy (SUDEP) and accidents (20–23).

Drug-refractory epilepsy is defined as the occurrence of uncontrolled seizures despite two tolerated and appropriately chosen antiepileptic drugs (AEDs) used either in combination or as monotherapies (11). Repeated changes in AEDs during disease course are a hallmark of drug-refractory epilepsy. Patterns of AED prescription changes were used to define patients with uncontrolled seizures in the database. In order to be able to study patients with severely drug-refractory epilepsy (SDRE), we chose patients who had used a minimum of four different AEDs in an 18-month period. This study’s purpose is the examination of long-term (3-year) follow-up, between 2008 and 2013, of SDRE patients. Data were sourced from the German Health Risk Institute (HRI) research database (24) containing details of four million Germans insured by statutory health insurance (Gesetzliche Krankenversicherung). This top-down approach allows for the examination of a high number of patients affected by epilepsy in Germany.

## Materials and Methods

This is a retrospective longitudinal analysis of secondary data carried out on the research database of the German HRI providing access to the details of approx. four million individuals (5% of the total population of Germany) covered by statutory health insurance (24). The sample was so designed as to be representative of the population of Germany by age and gender. The analysis proceeds from the perspective of costs that must be met by statutory health insurance in Germany. Data available were anonymous at the patient level, but included diagnosis, admissions as inpatients, practitioner consultations, medication used, and other items covering the use of a healthcare service. In Germany, physicians’ claims must be submitted at the end of each quarter, so that there are four time units for each year in the dataset and each of these units represents a period of 3 months. In total, 24 quarters in the insurance years 2008–2013 were available. The study was granted approval by the ethics committee of the University of Frankfurt. STROSA guidelines (Standardized Reporting Of Secondary data Analyses) were followed (25).

### Identification of Study Population Affected by SDRE

Records with codes for epilepsy (G40*) from the ICD-10-GM (10th revision of the International Statistical Classification of Diseases and Related Health Problems, German Modification, www.dimdi.de) were used to identify patients with epilepsy. Since, at the level of the third and fourth digits, epilepsy codes between the ICD-10 and ICD-10-GM systems show no discernible difference, ICD-10 is the term used throughout this article. The ICD-10 coding has already been used in Canada and Germany to identify cases of epilepsy and status epilepticus and demonstrated sensitivity and positive predictive value up to 98% (26–32).

Since no ICD-10 code exists for refractory epilepsy, we added to the need for a confirmed G40* diagnosis the prescription of at least four different AEDs matching ATC-codes N03A in an 18-month period sometime between 2009 and 2010. The reason for choosing a cutoff of four different AEDs was to reflect the definition of drug-refractoriness laid down by the ILAE, and to exclude patients who had become free from seizures following prescription of two AEDs in combination following the previous failure of one different drug, while the 18-month period was chosen as a sufficient time period for the titration of the dose of three AEDs (11, 12). These criteria for the definition of SDRE were put together in a preliminary retrospective analysis of records of epilepsy patients. For each insured person, the date of prescription of the fourth AED decided what the 3-year follow-up period would be, with the previous year acting as baseline. We did not determine an epilepsy syndrome on the basis of only ICD-10 diagnoses, due to the complications afforded by mixing classification of seizure with syndrome classification. In addition, no precise correspondence exists between the ICD-10 codes for epilepsy and the epilepsy syndrome and the International League Against Epilepsy classification of seizures, as defined in the 1980s, and nor is there such correspondence with the most recent concepts and terminologies for seizure and epilepsy classification that were revised in 2009 and 2017 (33–35).

### Cost Calculations

Cost evaluation applied a top-down approach of costs covered by statutory health insurance. Inpatient care costs were derived from the German Diagnosis Related Groups (G-DRG; www.g-drg.de), with costs calculated for the single baseline year and for the three follow-up years (i.e., 2011, 2012, and 2013). Inpatient costs were assumed to be specific to epilepsy where the primary ICD-10 was either G40 (epilepsy) or G41 (status epilepticus). Previous studies will provide more details of how costs were calculated (15, 18, 36).

### Statistical Analysis

To comply with regulations concerning data protection, management and analysis of all data was conducted by the use of anonymous patient codes. The number of admissions as an inpatient, the number of comorbidities, and the degree of mortality were all compared with a cohort that represented the general population matched by both age and gender at a ratio of 20:1 where *n* = 15,380. None of the matched patients had a diagnosis of epilepsy, to detect excess resource utilization, morbidity, and mortality. Age and gender distribution are provided in Figure S1 in Supplementary Material. Differences between SDRE patients and matched controls were assessed using chi-square tests, and Kaplan–Meier methodology was adopted for comparison for survival of SDRE patients with matched controls. *p*-Values were two-sided in all cases and were accepted as statistically significant <0.05. Since the study was planned to have an explorative nature, no further adjustment for multiple testing was performed.

## Results

### Identification of SDRE Patients

We identified 769 patients meeting our definition of SDRE. Of those, 54.6% were male, mean age was 42.3 years, standard deviation (SD) was 21.9, and 19% (*n* = 146) were either children or adolescents below the age of 18 years; 61.3% of patients (*n* = 471) were of working age and 19.8% (*n* = 152) were older than 65 years.

### Prescription Patterns

**Figure 1:**
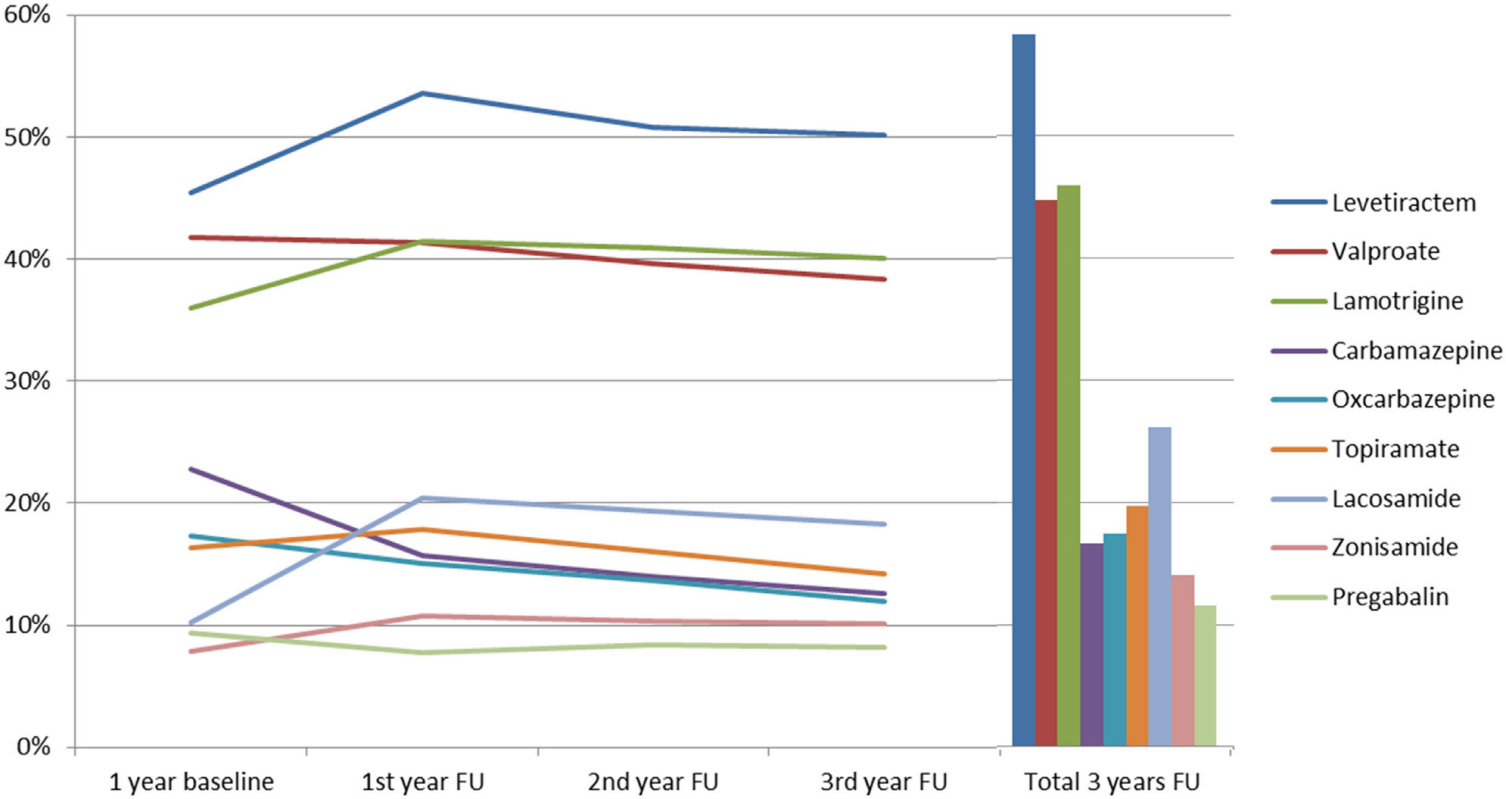
Prescription patterns of the anticonvulsants most used among severely drug-refractory epilepsy patients (FU, follow-up).

**Figure 2:**
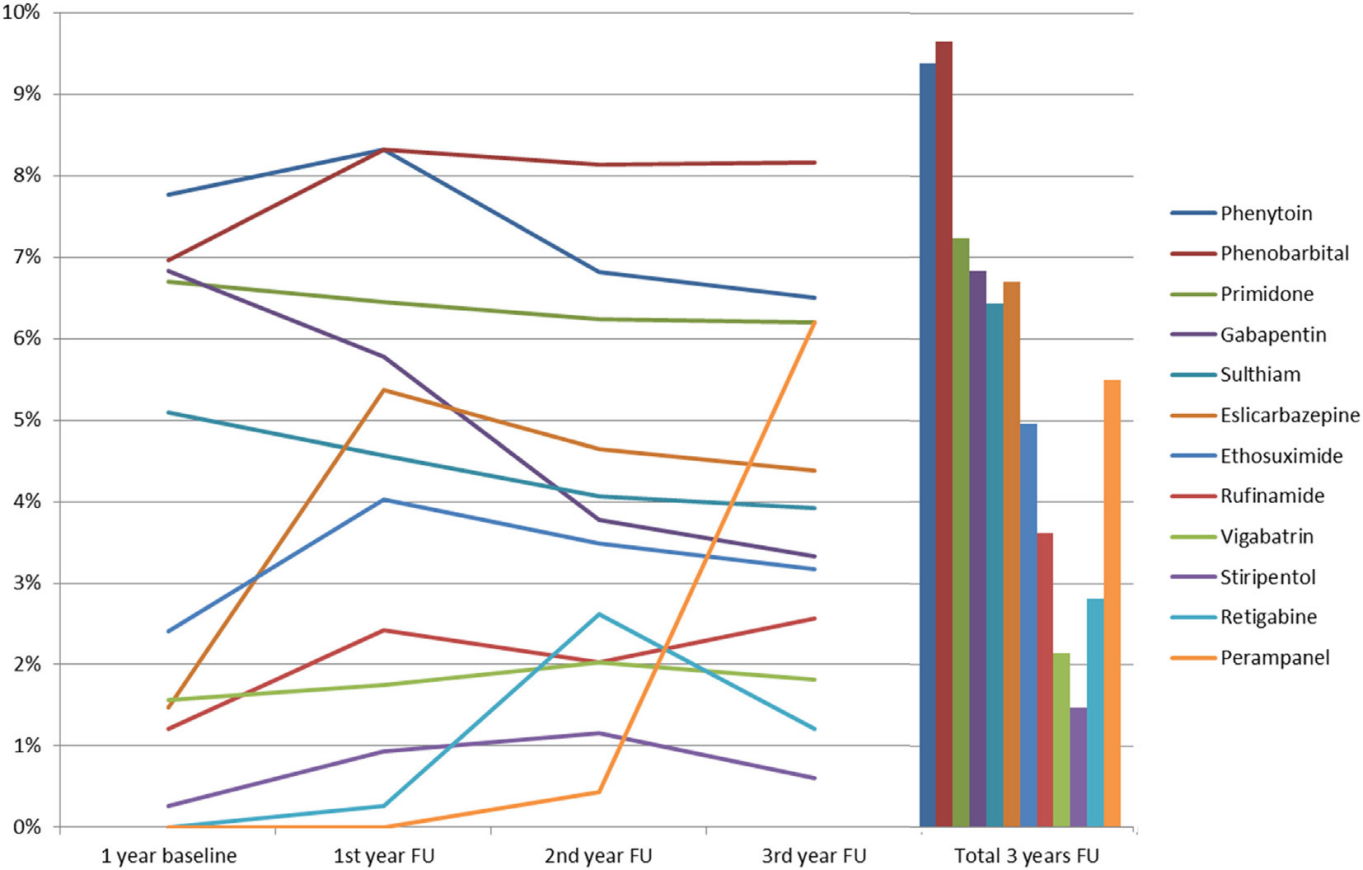
Prescription patterns of anticonvulsants of which <10% were used among severely drug-refractory epilepsy patients (FU, follow-up).

The time between prescription of the first three AEDs and prescription of the fourth AED varied with a mean latency of 212 days (SD 136.2). During the 3-year follow-up period, each patient received an average of 5.3 AEDs (SD 1.6), with the most commonly prescribed being: levetiracetam (56.6%, *n* = 435 during the 3 years of follow-up); lamotrigine (44.6%, *n* = 343); valproate (43.4%, *n* = 334); lacosamide (25.4%, *n* = 195); and topiramate (19.1%, *n* = 147). Further details are shown in Figure 1. More recently introduced AEDs, such as lacosamide, were prescribed twice during follow-up, as compared to baseline (*p* < 0.001); in contrast to that, carbamazepine (*p* < 0.001) and oxcarbazepine (*p* = 0.004) prescriptions fell significantly during follow-up. Perampanel was approved in 2012 and prescriptions increased from 0.4% of patients during the second follow-up year to 6.2% in the third follow-up year, which put this drug ahead of gabapentin, retigabine, and eslicarbazepine and showed a very rapid adoption rate. Further details are available in Figure 2.

Benzodiazepines were prescribed in a large percentage of patients, with lorazepam being prescribed at least once for 284 patients (36.9%) in the three follow-up years, followed by diazepam (*n* = 217, 28.2%), clobazam (*n* = 192; 25.0%), and clonazepam (*n* = 113; 14.7%).

Other drugs that were prescribed to SDRE patients at least once during the three follow-up years included: antibiotics (e.g., cefuroxime 20.5%); pain medication (e.g., ibuprofen 40.3%; metamizole 35.5%); antidepressants (citalopram 10.9%); proton pump inhibitors (pantoprazole 29.6%, omeprazole 20.4%); and neuroleptics (risperidone 7.9%; melperone 7.7%). Further information is contained in Table S1 in Supplementary Material.

### Hospitalization and Outpatient Visits

**Figure 3:**
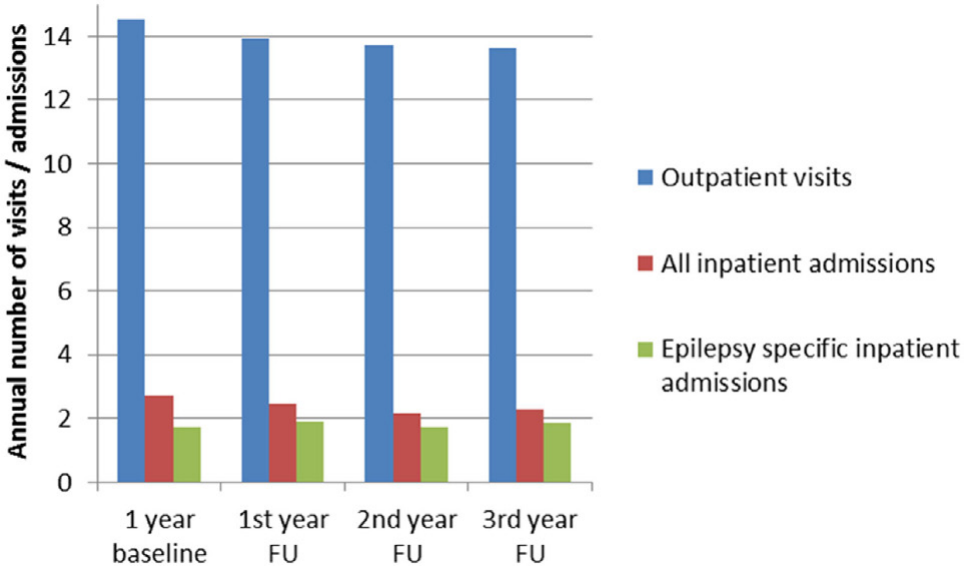
Annual inpatient admissions, epilepsy-specific admissions, and outpatient visits during baseline and FU in patients with epilepsy (FU, follow-up).

The overall number of patients who were admitted to hospital at least once during the three follow-up years was 568 (73.9%) (total admissions: 2,403; mean number of admissions per hospitalized patient: 4.2; total number of days in hospital, 23,346; mean stay 9.7 days). Of this total of 568, at least one admission was due to epilepsy in the case of 353 patients (total number of epilepsy-related admissions: 1,002, mean number of admissions per patient: 2.8; total number of days in hospital 10,474, mean stay 10.5 days). Figure 3 provides a summary of annual outpatient visits, annual overall admissions to hospital, and annual admissions to hospital for courses related to epilepsy.

Epilepsy-related hospital admissions per annum averaged from 1.7 to 1.9, and the epilepsy-related length of stay averaged from 10 to 11.1 days per annum. The rates of annual admission for complex treatment of epilepsy (“Komplexbehandlung Epilepsie” OPS 8-972.x) averaged from 6% to 6.8%, and at least one such complex treatment during the three follow-up years was provided to 13.6% (105/769) of patients. Annual rates of admission for non-invasive video-EEG-monitoring [OPS 1-210 (37)] averaged from 1.6% to 3.2%; 5.7% (44/769) of patients had at least one such non-invasive monitoring during the three follow-up years, while invasive monitoring was performed in five (0.6%) patients [OPS 1-211 (37)]. Brain surgery (OPS 5-010) was performed in 4% of the patients (31/769), while annual rates ranged between 1.5 and 2.1%. Within the three follow-up years, 94 patients (12.2%) were hospitalized as a result of a status epilepticus.

In the three follow-up years, annual rates of hospitalization ranged from 42.7% to 55%, which was a significant increase compared to the matched controls (11.6–12.8%, *p* < 0.001). Four thousand two hundred and ninety-seven (27.9%) of controls were hospitalized at least once in the three follow-up years; the total number of admissions among controls was 8,461 representing a mean number of admissions per matched hospitalized control of 1.97. Their total days spent in hospital amounted to 63,179, resulting in an average length of stay of 7.5 days.

Annual outpatients visits ranged between 13.6 and 14.0 contacts per year, see Figure 3. General practitioners were seen between 4.9 and 5.0 times per year by 94–96% of the SDRE patients. Neurologists were seen 2.9–3 times a year by 29–30% of the patients and neuropsychiatrists (Nervenarzt) were seen 3.4–3.5 times a year by 38–42% of the patients. Neuropediatricians were seen 2.1–2.2 times a year by 2.9–3.3% of the patients. Radiologists were seen 1.2–1.4 times a year by 19.6–23.2% of the patients. Prescription of AEDs was performed mainly by general practitioners (52–58% per year) ahead of neurologists (24–25% per year) and neuropsychiatrists (Nervenarzt: 35–36% per year).

### Comorbidities and Mortality

**Figure 4:**
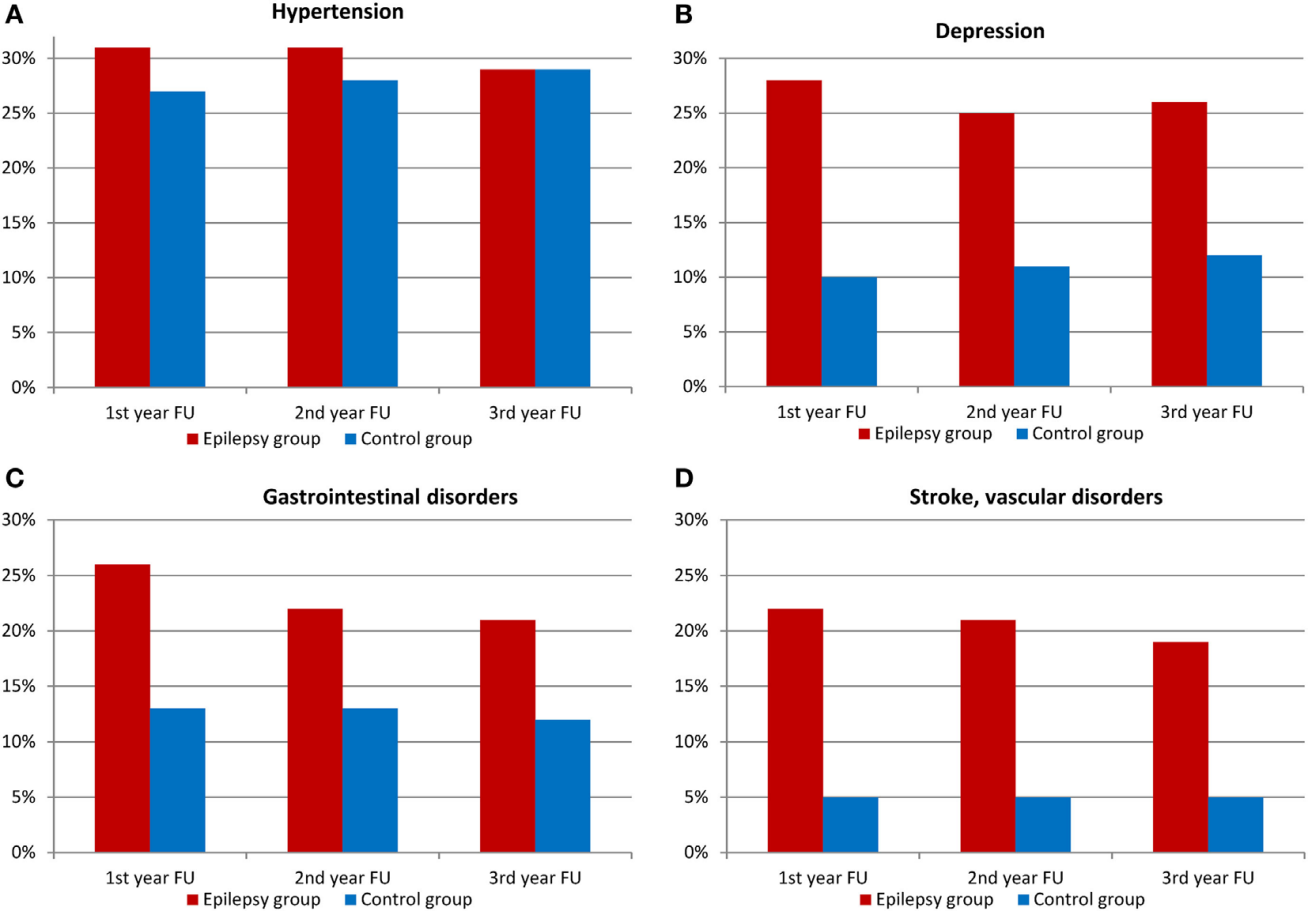
Comorbidities among patients with epilepsy and control group (FU, follow-up). **(A)** Hypertension, **(B)** depression, **(C)** gastrointestinal disorders, and **(D)** stroke, vascular disorders.

The number of comorbidities in the SDRE group was significantly higher than in the control group, see Figure 4. Depression was diagnosed in 28% in the first year of follow-up compared to 10% in the general population (*p* < 0.001). Further psychiatric comorbidities comprised organic mental disorders (ICD-10 F06) in 24.8%, somatoform disorders (ICD-10 F45) in 20.8%, and personality and behavioral disorders (ICD-10 F07) in 19.6%.

**Figure 5:**
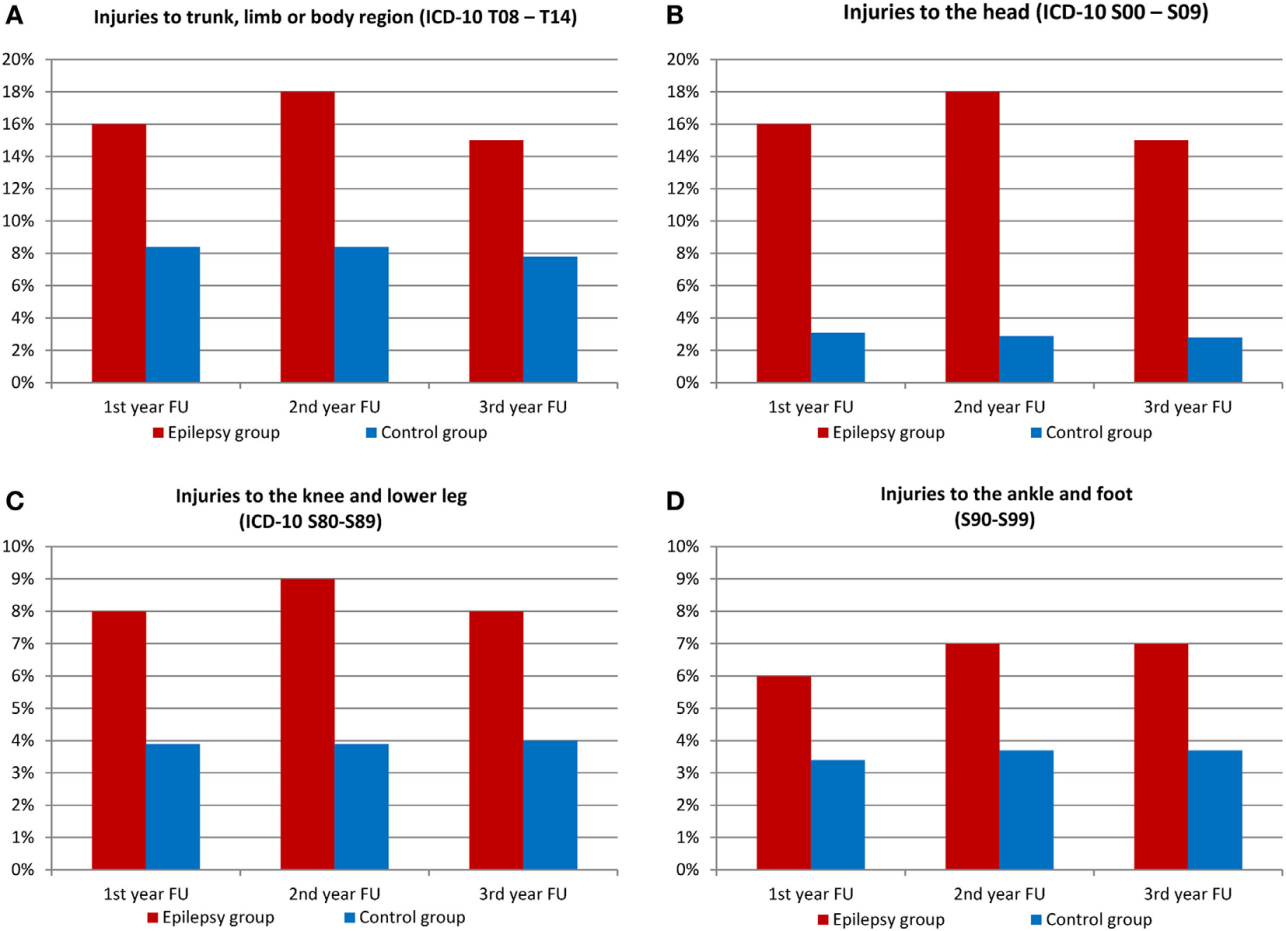
Injuries among patients with epilepsy and control group (FU, follow-up). **(A)** Injuries to trunk, limb, or body region (ICD-10 T08–T14), **(B)** injuries to the head (ICD-10 S00–S09), **(C)** injuries to the knee and lower leg (ICD-10 S80–S89), and **(D)** injuries to the ankle and foot (S90–S99).

Other conditions were gastrointestinal disorders (26 against 13%, *p* < 0.001) and vascular disorders (22 vs. 5%, *p* < 0.001). As Figure 4 shows, hypertension distribution showed no difference. SDRE patients were also more likely to suffer injuries, with 16% suffering head injuries compared with 3% of the control cohort (*p* < 0.001) and injuries to trunk and limbs also at 16%, compared, in this case, with 8% in general population (*p* < 0.001), please refer to Figure 5 for details. Table S2 in Supplementary Material shows the details of ICD-10 coding in inpatient and outpatient settings during the three follow-up years.

**Figure 6:**
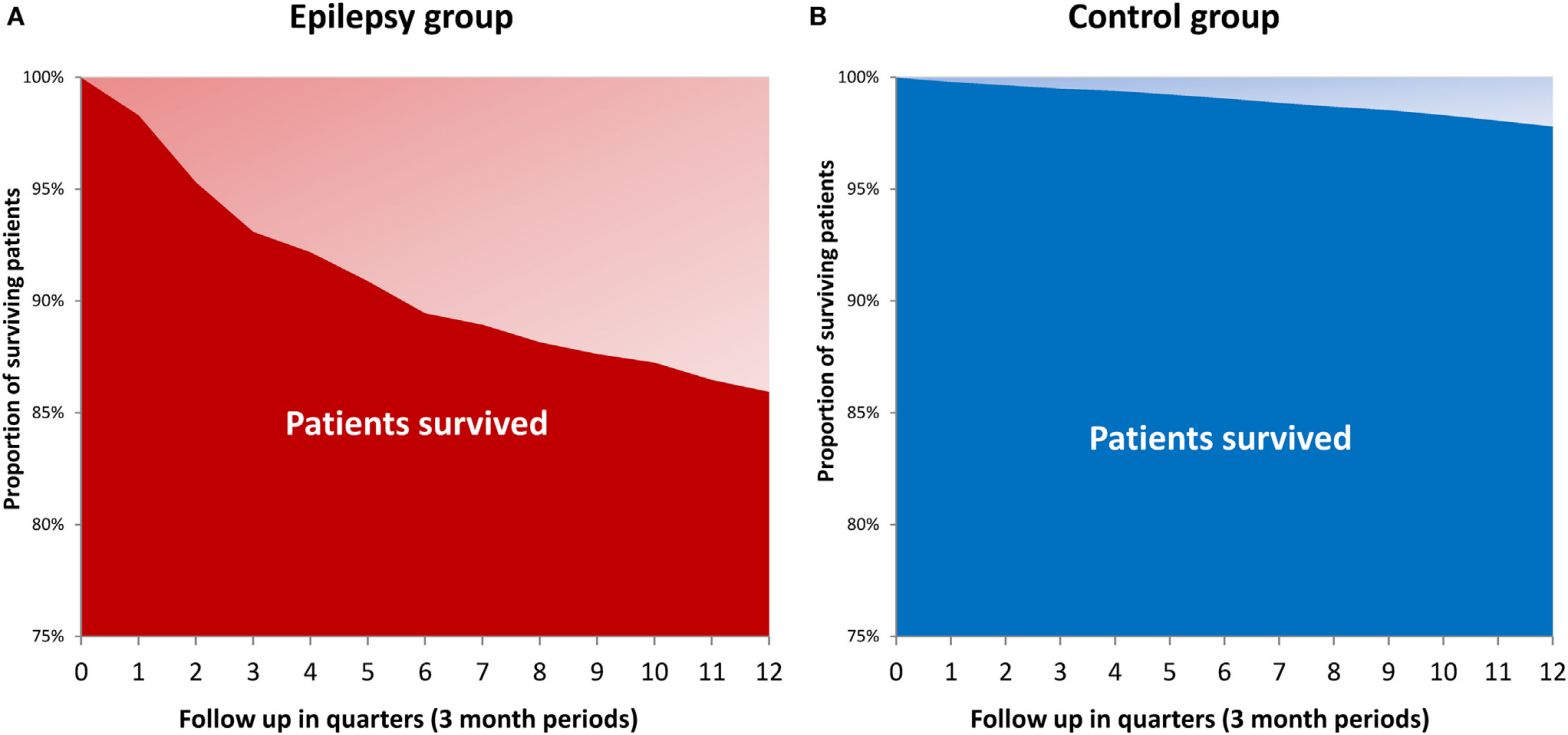
Mortality among patients with epilepsy **(A)** and control group **(B)**.

Mortality rate of SDRE within 3 years was 14%, compared with the matched cohort’s 2.1% (*p* < 0.001). Figure 6 uses the Kaplan–Meier method to show survival times. SDRE patients’ mortality rate was at its highest (7.8%) during the first follow-up year.

### Costs

**Figure 7:**
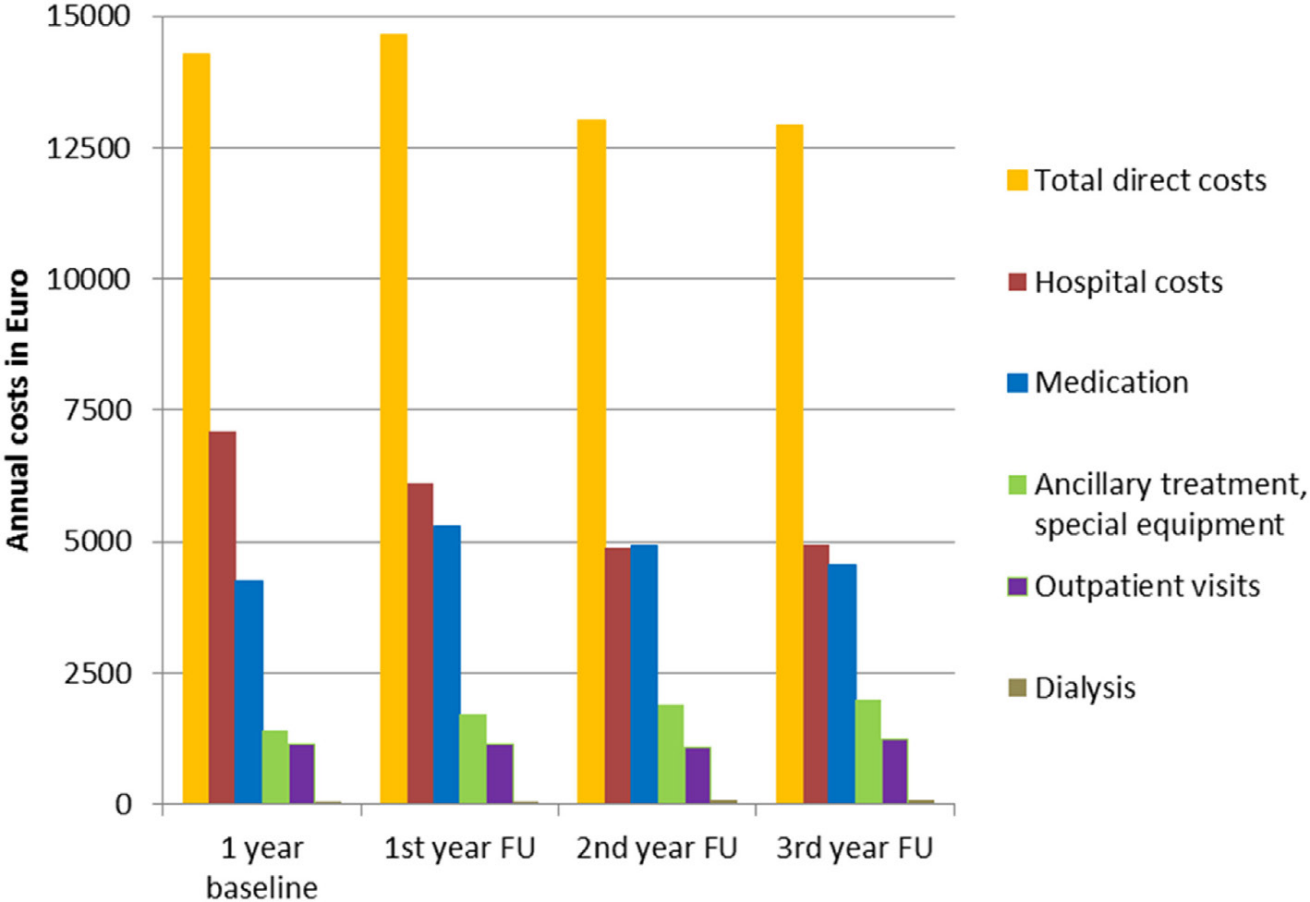
Annual inpatient treatment, medication, ancillary treatment, special equipment, outpatient visits, and dialysis costs, separately and as total direct costs.

The annual direct costs of treating SDRE patients totaled to between €12,925 and €14,639 during the three follow-up years. The annual inpatient treatment costs were between €4,880 (37% of total direct costs) and €6,110 (42%). Annual medication costs were between €4,565 (35%) and €5,294 (38%). Figure 7 gives details of the costs, both at the baseline year and in follow-up years.

## Discussion

This is the first nationwide study using health insurance data analyzing the long-term treatment patterns, costs, and mortality of SDRE patients in Germany. It shows the high burden imposed on SDRE patients in the form of higher consumption of resources, comorbidities, injuries, and mortality when compared to matched cohort drawn from the population at large.

Prescription patterns for 2011, 2012, and 2013 from this study demonstrate an increased use of newer anticonvulsants. The study has also confirmed findings from other studies on prescription patterns in drug-refractory patients who used a top-down approach (38–40). Comparison of these prescription patterns with previous German studies carried out in 2003 (15), 2008 (18), 2009 (38), 2011 (8, 41), and 2012 (9) show a significant increase in the use of “newer” AEDs and a marked reduction in enzyme-inducing anticonvulsant prescriptions. Since 2008, German guidelines recommend that the first choice for monotherapy in focal epilepsy should be lamotrigine or levetiracetam and that anticonvulsants with drug–drug interactions should not be used. Recent studies show that German prescription patterns for anticonvulsants follow these recommendations with marked reduction in prescription of carbamazepine and phenytoin, that is largely in line with the current guidelines (9, 31, 42). Benzodiazepines were used commonly in our SDRE cohort; however, we cannot differentiate in detail which benzodiazepines were prescribed for rescue therapy, ongoing anticonvulsive treatment, or use in depression or anxiety disorders.

Our study suggests a possible undertreatment of SDRE patients. Only 13.6% of patients had specialized inpatient epilepsy treatment, and the proportion of patients admitted to presurgical evaluation through video-EEG-monitoring was 5.7%, even fewer (37, 43). A substantial proportion of 30–40% of the patients did not attend a neurologist or neuropsychiatrist during each year of follow-up. Despite the AED polytherapy in these patients, anticonvulsive therapy was not prescribed by a neurologist or neuropsychiatrist. It can be inferred from these low percentages that therapeutic and diagnostic options may be underused. Due to the underutilization of specialist care, some patients might have suffered from a SDRE. There are difficulties in gaining access to epilepsy centers and surgery programs for epilepsy; this seems in line with other studies that have shown that referral takes 15–20 years on average (44–48). Evaluation at an epilepsy center of patients who had been referred showed that only 30% came with magnetic resonance imaging (MRI) that conformed to the guidelines and was sufficient, whereas this was not the case in 70% of the referred patients. In 10%, MRI had not been carried out (49). Studies to be carried out in future should also follow-up treatment of SDRE patients and should pay particular attention to such aspects of the guidelines as: what information about their disease has been provided to patients (48, 50); and what access refractory patients have to the comprehensive care that epilepsy centers can provide or to the provision of epilepsy nurses and/or epilepsy counseling services to patients with a low access threshold (51, 52).

The mortality rate of SDRE in patients within 3 years was 14%, which is seven times the rate for a control population matched for age and gender. The increased mortality is high and in line with a recently published, matched nationwide study from Denmark reporting mortality among epilepsy patients of more than 10% 3 years after diagnosis of epilepsy (53). Causes of death among people with epilepsy are manifold. The risk of a SUDEP is up to 9.3 per 1,000 person-years in a refractory population (54). Given such SUDEP risk, we would expect a crude number of around 18–20 SUDEP cases during the 3 years of follow-up in our cohort, which covers approximately 2,200 person-years. Studies of SUDEP lifetime risks have shown that, even in an unselected epilepsy patient cohort, onset at one year of age leads to an 8% SUDEP risk by age 70. That risk falls to 7.2% if the epilepsy began at 15 years of age, and to 4.6% for a starting age of 30 years (23). A study carried out in Finland on long-term mortality rates in epilepsy that begins in childhood followed 245 patients for 40 years (20). A total of 60 patients (24%) died, which was three times the rate that would be expected in a cohort from the general population adjusted for age and gender. Active epilepsy and a remote symptomatic cause correlated with mortality. In total, 33 deaths (55%) were epilepsy-related, with SUDEP present in 18 cases (30%), seizure (either definite or likely) in 9 (15%), accidental drowning in 6 (10%), and status epilepticus in 4 (7%). Mortality resulting either from injuries or from status epilepticus might also suggest an explanation of mortality in our study, which shows an increase in the number of injuries and that status epilepticus was responsible for 12.2% of the epilepsy population being hospitalized. There is a mean mortality association between 15 and 20% with status epilepticus (32, 55–57). The high number of injuries appearing in our study confirms the results of previous studies (58–61). An American study of 52 patients suffering from refractory focal epilepsy showed that injury in 21% of temporal lobe epilepsies and 8% in extratemporal epilepsies had occurred in the year previous to the survey (61). Temporal lobe epilepsy showed a 57% lifetime prevalence of injuries with a 22-year average duration, while, for extratemporal epilepsies, this lifetime prevalence was 17% and the duration of the disease 17 years. Comparing interviews with patient records showed that injuries were documented in only 45% of cases and that whether documentation occurred depended on how severe the injuries were (61). A prospective European study on disease and injury used friends and relatives as a control for 12 years (58) and found that the likelihood of accident and injury in the epilepsy patients was 21%, compared with 14% for the controls (58). There was a correlation between number of injuries and seizure frequency (58). The continuing significance of injuries caused by seizures as a serious and persistent problem in cases of childhood onset epilepsy was confirmed by a population-based study conducted by the Nova Scotia Childhood Epilepsy cohort (59). In a follow-up averaging 24 years, 11% of patients (52/472) experienced one or more serious injuries, with the total of injuries being 81. Of these, the most frequently reported were lacerations requiring sutures (30%), fractures (19%), broken teeth (14%), concussions (10%), and burns (5%). Also reported were one drowning that proved fatal, two near-drownings, one severe eye injury, and three dislocations of the shoulder. As before, the risk factors were symptomatic generalized epilepsy and intractable epilepsy. Most injuries occurred during the patient’s normal daily activities. They occurred at any stage in the patient’s life and were judged as not easily preventable. In line with our results on vascular and gastrointestinal disorders, studies on somatic comorbidities in epilepsy patients show an increase in stomach/intestinal ulcers, stroke, urinary incontinence, bowel disorders, migraine, Alzheimer’s disease, and chronic fatigue (62, 63). Overall, our study’s high mortality figures can, therefore, be seen to confirm the findings of previous studies (20, 53). A significant proportion may be related to epilepsy as a result of SUDEP, status epilepticus, or injuries.

Our results show that the total direct costs of treatment of SDRE patients amounted to between €12,925 and €14,639 per annum in the three follow-up years, exceeding the annual average cost of €3,011 for any insurant in Germany (64). The main constituents of the direct cost were inpatient costs, amounting to 37%, 42% of the total direct costs, and cost of medication, which came to between 35 and 38%; these percentages did not change over time, which demonstrates that there is a need for increased levels of healthcare services for SDRE patients. Studies of cost of illness (2) show hospitalization costs to be less subject to change than medication costs. Hospitalization costs continue to be a major element of total costs, and this confirms the results of other top-down studies from Denmark (65) and the United States of America (66, 67). Evaluating large cohorts, as has been the case with this study, allows data to be captured from cost-intensive patients presenting infrequently to hospital [as, for example, with status epilepticus (68–70) or for video-EEG monitoring or epilepsy surgery (71)]. Smaller, bottom-up studies are more likely to overlook these patients.

While this study was designed with great care, it nevertheless has limitations of the sort such studies cannot escape. Top-down studies are able to include all epilepsy patients, regardless of whether they could take part in field studies, but there is still difficulty in identifying mortality and injury as definitely caused by or connected to epilepsy. Mortality can be due to underlying causes, such as stroke, traumatic brain injury, or malignancies. Difficulties also remain in the categorization of costs between, for example, costs directly related to epilepsy and costs connected with comorbidities. Even though the control group was age and gender matched, it would have been useful to have a match by propensity score so that the impact of comorbidity could be minimized (72). It is possible to explore what impact or influence epilepsy and anticonvulsive therapy have on such comorbidities as osteoporosis and depression, both of which, whether separately or in combination, have the potential to increase mortality, injury frequency, and consumption of resources. It is also possible that our results have been affected by matters of methodology and/or by the SDRE definition used. These possible effects include the origin of the information from health insurance database, since definitive patient information at the chart level is not available from such records due to data protection rules. AEDs might have been discontinued due to adverse events and not due to refractoriness. Therefore, we cannot provide sensitivity and specificity values for the definition used. Classifying SDRE by means of an algorithm based on treatment is perhaps insufficiently precise.

## Conclusion

This study’s significance arises from analysis of health insurance data based on a sample population for longitudinal analysis of SDRE patients; this is the first time that this has been done. Future analysis should explore ways in which new approaches to treatment could improve outcomes for severely affected patients.

## Ethics Statement

This study was carried out in accordance with the Declaration of Helsinki. As this study used anonymous insurance data written informed consent was not obtained. The protocol was approved by the ethics committee of the University of Frankfurt. We confirm that we have read Frontiers in Neurology position on issues involved in ethical publication and affirm that this report is consistent with these guidelines.

## Author Contributions

AS and CG generated the research idea and concept. WL acquired and analyzed the data. AS, CG, WL, FR, and JPR made critical revisions for important intellectual content and interpreted the data. AS wrote the manuscript. AS, CG, WL, FR, and JPR approved the final manuscript.

## Conflict of Interest Statement

AS has received support and honoraria from Bayer HealthCare, Boehringer Ingelheim, Desitin Arzneimittel, Eisai, LivaNova, Pfizer, Sage Therapeutics, UCB Pharma and Zogenix. CG is an employee of LivaNova Deutschland GmbH, München; Germany. WL is an employee of HGC GesundheitsConsult GmbH, Düsseldorf, Germany. FR reports grants and personal fees from Eisai, UCB Pharma, Desitin Arzneimittel, Novartis, Medtronic, Cerbomed, ViroPharma, Sandoz, Sage Therapeutics and Shire, grants from European Union and Deutsche Forschungsgemeinschaft. JPR reports grants from Lundbeck. The reviewer RD and handling editor declared their shared affiliation.
