# Supplementary material for: The Burden of Severely Drug-Refractory Epilepsy: A Comparative Longitudinal Evaluation of Mortality, Morbidity, Resource Use, and Cost Using German Health Insurance Data
Source: Front Neurol. 2017 Dec 22;8:712. doi: 10.3389/fneur.2017.00712 (PMC5743903; doi:10.3389/fneur.2017.00712)
Supplement: Supplementary file 1 [file Data_Sheet_1.docx]

APPENDIX

Supplementary Figure S1a Histogram of age and gender distribution among epilepsy patients


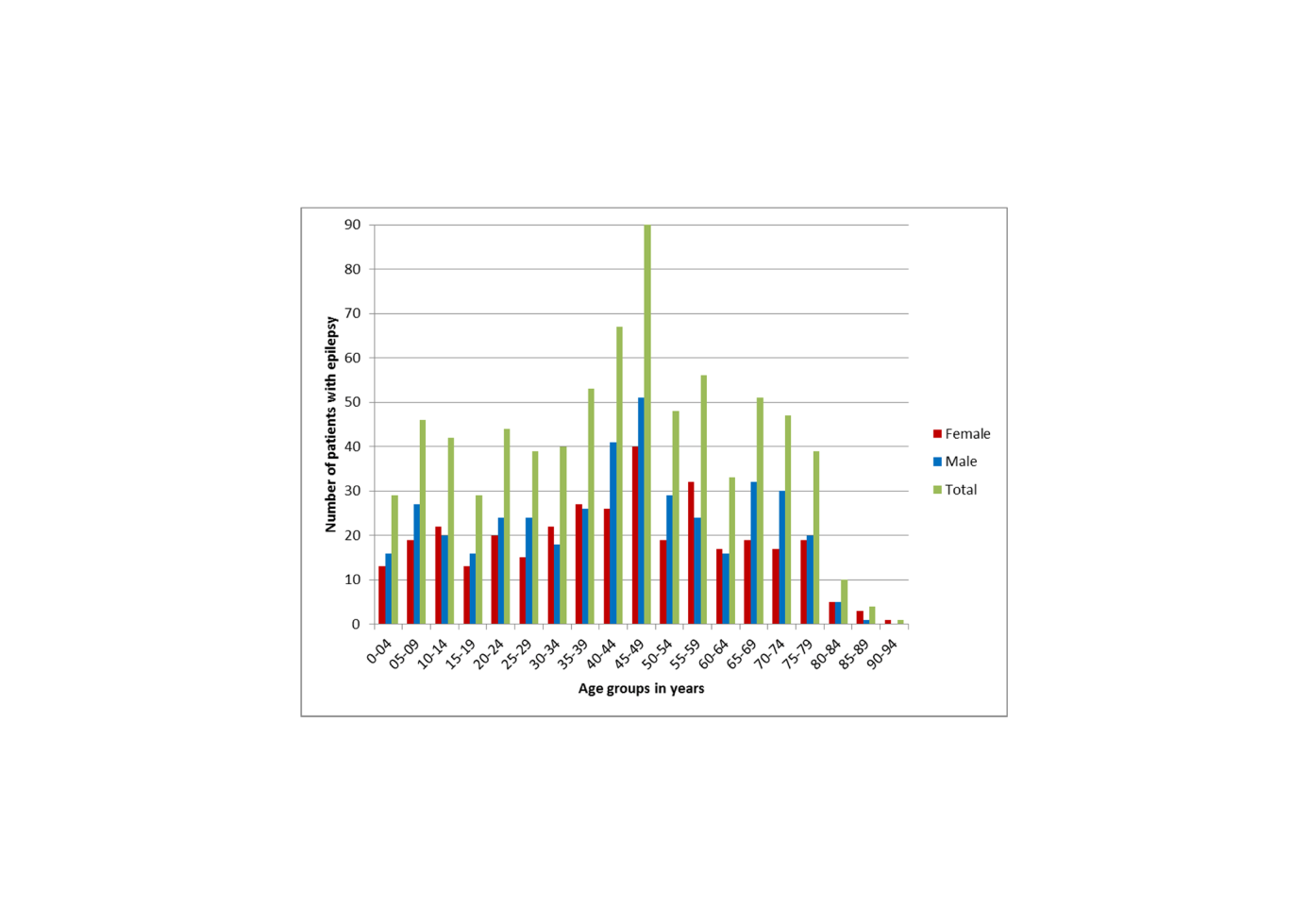


Supplementary Figure S1b Histogram of age and gender distribution among matched controls


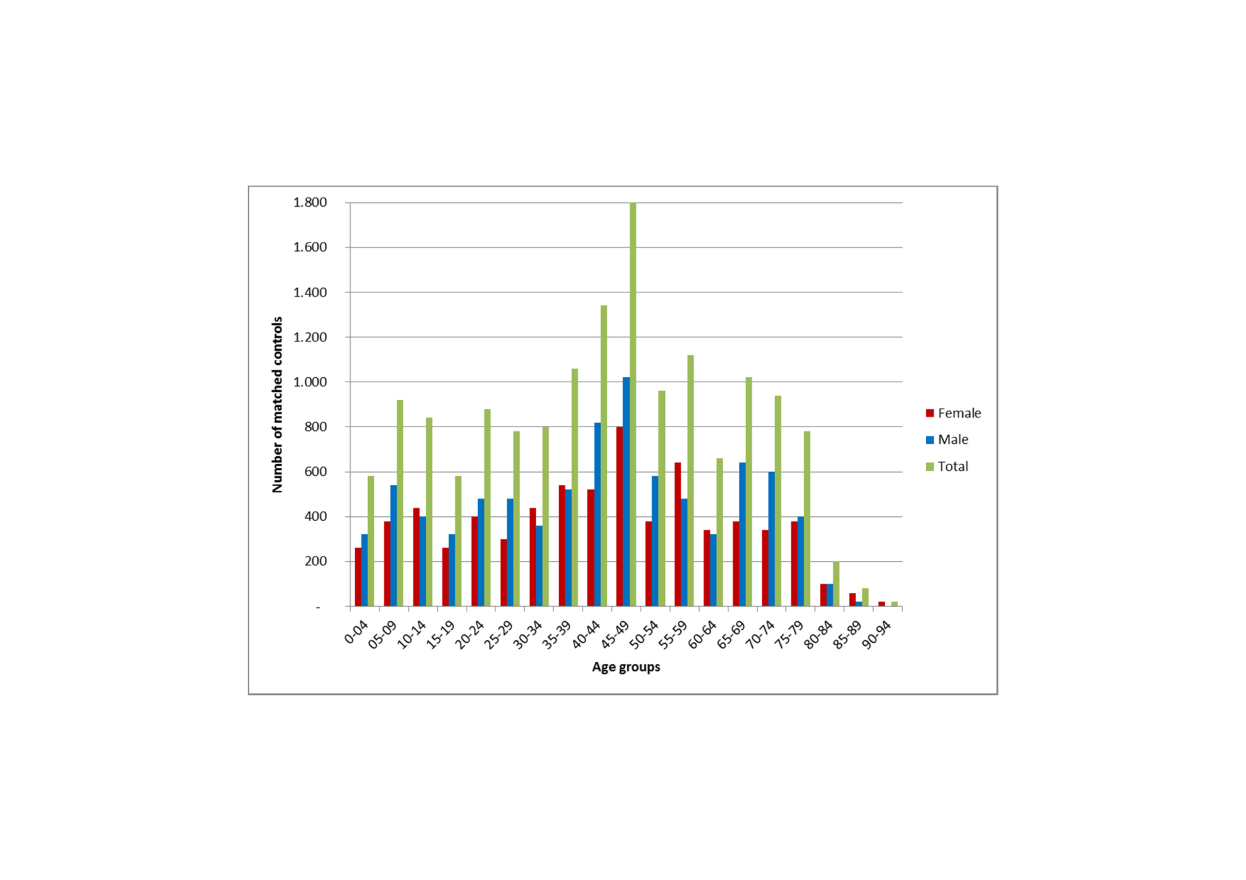


Supplementary Table 1 Overall comedication in epilepsy patients during follow-up of three years

| **Medication** | Number  of patients | % |
| --- | --- | --- |
| **Pain medication** |  |  |
| Ibuprofen | 310 | 40.3% |
| Metamizole | 273 | 35.5% |
| Diclofenac | 151 | 19.6% |
| Paracetamol | 78 | 10.1% |
| **Proton pump inhibitors** |  |  |
| Pantoprazole | 228 | 29.6% |
| Omeprazole | 157 | 20.4% |
| **Antibiotics** |  |  |
| Cefuroxime | 158 | 20.5% |
| Amoxicilline | 150 | 19.5% |
| Ciprofloxacin | 120 | 15.6% |
| Sulfamethoxazole and Trimethoprim | 96 | 12.5% |
| Cefaclor | 69 | 9.0% |
| Amoxicillin and ß-lactamase inhibitors | 68 | 8.8% |
| Doxycycline | 66 | 8.6% |
| Clindamycin | 65 | 8.5% |
| Azithromycin | 59 | 7.7% |
| **Antidepressents and neuroleptics** |  |  |
| Citalopram | 84 | 10.9% |
| Risperidone | 61 | 7.9% |
| Melperone | 59 | 7.7% |
| **Other** |  |  |
| Metoclopramide | 120 | 15.6% |
| Levothyroxine | 109 | 14.2% |
| Salbutamol | 85 | 11.1% |
| Prednisolone | 78 | 10.1% |
| Simvastatin | 75 | 9.8% |
| Acetylsalicylic acid | 71 | 9.2% |
| Colecalciferol | 70 | 9.1% |
| Metoprolol | 68 | 8.8% |
| Torasemid | 65 | 8.5% |

Supplementary Table 2 Overall comorbidities coded in inpatient and outpatient setting in epilepsy patients during follow-up of three years

| **ICD-Code** | **Disease** | **Number  of patients** | **%** |
| --- | --- | --- | --- |
| I10 | Essential hypertension | 281 | 36.5% |
| N39 | Diseases of the urinary system | 277 | 36.0% |
| J06 | Acute infections of upper respiratory tract | 275 | 35.8% |
| M54 | Back pain | 266 | 34.6% |
| F32 | Depressive episode | 229 | 29.8% |
| T14 | Injury in an unspecified body region | 223 | 29.0% |
| R32 | Urinary incontinence | 217 | 28.2% |
| E87 | Disorders of water and electrolyte balance as well as acid-base balance | 203 | 26.4% |
| E78 | Dyslipidaemia | 202 | 26.3% |
| F06 | Mental disorders due to injury or dysfunction of the brain or a physical disorder | 191 | 24.8% |
| H53 | Visual disturbances | 189 | 24.6% |
| L30 | Dermatitis | 187 | 24.3% |
| J20 | Acute bronchitis | 176 | 22.9% |
| R10 | Abdominal and pelvic pain | 171 | 22.2% |
| F79 | Cognitive impairment | 163 | 21.2% |
| R26 | Disruption of gait and mobility | 161 | 20.9% |
| F45 | Somatoform disorders | 160 | 20.8% |
| Z74 | Need of long-term care | 158 | 20.5% |
| F07 | Personality and behavioral disorder due to illness, injury or function disorder of the brain | 151 | 19.6% |
| G81 | Hemiparesis and hemiplegia | 148 | 19.2% |
